# *Clonorchis sinensis*-driven hepatocarcinogenesis via E2F1-CD24 transcriptional axis: mechanistic and therapeutic implications

**DOI:** 10.1186/s13071-025-06979-6

**Published:** 2025-08-19

**Authors:** Wen-Min Lu, Jin Yan, Zhao-Ji Liu, Yong Wu, Qian-Ru Cui, Ji Feng, Yu Chen, Guang-Zhi Zhu, Tao Peng, Jing Zhou, Guo-Dong Lu

**Affiliations:** 1 Department of Toxicology, School of Public Health, Guangxi Medical University, Nanning, Guangxi China; 2 School of Public Health, Fudan University, Shanghai, China; 3 Department of Physiology, School of Basic Medical Sciences, Guangxi Medical University, Nanning, Guangxi China; 4 Zhongshan School of Medicine, Sun Yat-Sen University, Guangzhou, Guangdong China; 5 Department of Pathology, National Cancer Center/National Clinical Research Center for Cancer/Cancer Hospital and Shenzhen Hospital, Chinese Academy of Medical Sciences and Peking Union Medical College, Shenzhen, Guangdong China; 6 Hengzhou Center for Disease Control and Prevention, Hengzhou, Guangxi China; 7 Department of Hepatobiliary Surgery, The First Affiliated Hospital of Guangxi Medical University, Nanning, Guangxi China

**Keywords:** Hepatocellular carcinoma, *Clonorchis sinensis*, CD24, E2F1, Tumor promotion

## Abstract

**Background:**

Hepatocellular carcinoma (HCC) remains a global health burden, with disproportionately high mortality in China’s Guangxi region, where endemic *Clonorchis sinensis* (*C. sinensis*) infection coincides with elevated HCC incidence. Preliminary single-cell sequencing revealed marked overexpression of cluster of differentiation 24 (CD24) in HCC tissues, suggesting its potential pathological role. This study aims to elucidate the oncogenic mechanisms of *C. sinensis* excretory-secretory products (CsESPs) and their link to CD24-mediated HCC progression.

**Methods:**

We employed an integrated clinical and experimental approach. First, clinical cohort analysis assessed CD24 expression in *C. sinensis*-associated HCC cases. Multiplatform bioinformatics validation (GEPIA/UALCAN/TIMER) evaluated CD24’s prognostic significance and immune microenvironment modulation. Functional studies (quantitative polymerase chain reaction (qPCR), Western blotting, CCK-8 assays, flow cytometry) examined CsESPs’ effects on CD24 expression, cell proliferation, and apoptosis. Mechanistic investigations (chromatin immunoprecipitation, dual-luciferase reporter assays) identified E2F1-mediated transcriptional activation of CD24. siRNA-mediated CD24 knockdown validated its role in CsESPs-driven oncogenesis. Additionally, the expression of immune checkpoint (CTLA-4, LAG-3) was assessed in the co-cultures of peripheral blood mononuclear cells (PBMCs)–HCC cells.

**Results:**

Clinical cohort analysis confirmed significant CD24 upregulation in HCC, particularly in *C. sinensis*-infected cases. Bioinformatic analyses linked high CD24 expression to poor prognosis and immune microenvironment alterations. Functional assays demonstrated that CsESPs enhance CD24 expression, promoting proliferation and inhibiting apoptosis. Mechanistically, E2F1 directly binds to CD24 promoter, driving its transcription upon CsESPs exposure. CD24 silencing reversed CsESPs-induced oncogenic effects. Furthermore, CsESPs upregulated immune checkpoints (CTLA-4, LAG-3) in the co-cultures of PBMC–HCC cells, an effect reversed by CD24 knockdown.

**Conclusions:**

Our findings establish a novel parasitic carcinogenesis paradigm wherein *C. sinensis* promotes HCC development through E2F1-mediated transcriptional activation of CD24, simultaneously identifying prognostic biomarkers and therapeutic targets while suggesting combinatory immunotherapy strategies for parasite-associated HCC.

**Graphical Abstract:**

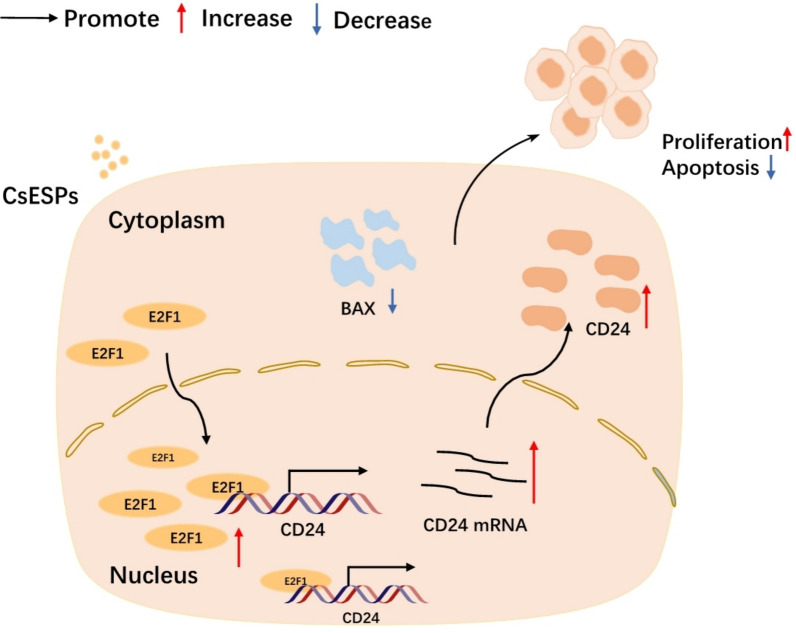

**Supplementary Information:**

The online version contains supplementary material available at 10.1186/s13071-025-06979-6.

## Background

Globally, liver cancer constitutes the third most lethal malignancy and sixth most frequently diagnosed cancer according to 2022 International Agency for Research on Cancer (IARC) statistics [1]. Chinese epidemiological surveillance demonstrates concordance, with liver cancer representing the second leading cause of cancer-related mortality and fourth most diagnosed malignancy nationally [2]. Among the various subtypes of liver cancer, hepatocellular carcinoma (HCC, referred to as liver hepatocellular carcinoma (LIHC) in database) is the most prevalent, accounting for approximately 85% of cases [3].

While nationwide hepatitis B virus (HBV) vaccination initiatives and aflatoxin control have driven progressive reductions in HCC incidence and mortality over two decades, significant regional disparities persist [4, 5]. National cancer registry data identify Guangxi province as exhibiting China’s highest HCC mortality rates, with gender-specific rates of 69.0/100,000 (male) and 17.8/100,000 (female)—markedly exceeding national averages of 36.5/100,000 and 12.8/100,000, respectively [6]. Notably, Guangxi demonstrates paradoxical upward trends in HCC incidence (annual percentage change +5.38%) and mortality (+9.23%) during 2010–2016 [7], indicating distinct etiological factors beyond conventional HBV- or aflatoxin-associated hepatocarcinogenesis. Of particular epidemiological significance, this region demonstrates China’s highest prevalence of endemic *Clonorchis sinensis* (*C. sinensis*) infection (6.68%) in China [8], strongly associated with local dietary practices of raw fish consumption. The persistent geographic overlap between exceptional HCC mortality burdens and helminthic infection prevalence implies potential proto-oncogenic interactions warranting mechanistic investigation.

*C. sinensis*, commonly known as the liver fluke, is a food-borne parasite prevalent in China, Korea, Northern Vietnam, and the Russian Far East [9]. Human infection occurs through the consumption of raw or undercooked freshwater fish containing *C. sinensis* metacercariae. Chronic *C. sinensis* infection induces progressive hepatobiliary pathology including biliary hyperplasia, periductal fibrosis, and cholangiocarcinoma development through sustained inflammatory responses [10, 11]. With 15 million global cases (87% in China) [12], this IARC group 1 carcinogen [13] demonstrates increasing oncogenic evidence in liver cancer pathogenesis. Recent clinical investigations revealed that *C. sinensis* co-infection with HBV accelerates HCC progression by enhancing cancer stem cell properties, serving as an independent prognostic factor for reduced overall survival (OS) (5-year OS: 47.8% versus 63.8% in HBV alone-infected cohorts; *n* = 946) [14, 15]. Experimental models demonstrate that infection promotes hepatic progenitor cell proliferation [16] and induces M1-like macrophage polarization via extracellular vesicle-derived Csi-let-7a-5p, mediating biliary injury through Socs1/Clec7a-dependent NF-*κ*B activation [17]. Other mechanistical studies revealed that the *C. sinensis* excretory–secretory protein CsGRN drives malignant transformation through EGFR-mediated RAS/MAPK/ERK and PI3K/AKT pathway activation in hepatocytes [18, 19], with complementary studies confirming its transformative effects on human intrahepatic cholangiocytes in vitro and biliary injury in vivo [20].

Building upon these findings, our preliminary single-cell sequencing data identify CD24 overexpression in *C. sinensis*-associated HCC specimens (unpublished observations). CD24, commonly referred to as the heat-stable antigen, is a surface protein anchored by glycosylphosphatidylinositol [21, 22]. It is overexpressed in various cancers and is implicated in regulating cancer cell proliferation, invasion, and immune evasion [23–25]. For instance, in ovarian and breast cancers, CD24 engages with Siglec-10 found on tumor-associated macrophages, thereby promoting immune evasion [21]. In liver, CD24 is absent in normally differentiated hepatocytes but detectable in hepatic progenitor cells (oval cells), suggesting a potential association with hepatic stem cell characteristics [26]. Notably, CD24 is overexpressed in HCC, where its expression correlates with increased tumor invasiveness, metastatic potential, enhanced proliferation, and activation of the Wnt/*β*-catenin signaling pathway [27]. However, the role of CD24 in the pathogenesis of HCC following *C. sinensis* infection remains poorly understood.

Given the potential link between *C. sinensis* infection and HCC progression, our objective was to explore the underlying mechanisms through which CsESPs influence CD24 expression and function in HCC cells and co-cultured immunocytes.

## Methods

### Clinical sample collection

Human primary HCC tissues and paired normal tissues were collected from September 2020 to December 2022 at the First Affiliated Hospital of Guangxi Medical University. All patients with HCC underwent surgical operation. Informed consent was acquired from all participants, and the research received approval from the Ethics Committee at the First Affiliated Hospital of Guangxi Medical University (ethics approval no.: 2024-E580-01). The eligibility criteria for patients with HCC in this study were delineated as follows. Inclusion criteria: (1) patients scheduled to undergo initial radical surgical resection as their primary therapeutic intervention, (2) patients with histopathologically confirmed HCC on the basis of postoperative tissue analysis, and (3) patients with comprehensive baseline demographic, clinical, and radiological data, along with complete follow-up records. Exclusion criteria: (1) patients undergoing palliative surgical procedures rather than curative-intent resection; (2) patients diagnosed with non-HCC malignancies, including intrahepatic cholangiocarcinoma, hilar/extrahepatic cholangiocarcinoma, metastatic hepatic lesions, combined hepatocellular-cholangiocarcinoma (HCC-ICC), tumors of indeterminate origin, or extrahepatic primary malignancies; (3) patients with recurrent HCC following prior treatment; (4) patients who received neoadjuvant therapies before surgery, encompassing systemic chemotherapy, radiotherapy, locoregional ablative treatments, immunotherapy, or molecular-targeted agents; and (5) patients with incomplete clinical documentation or missing pathological specimens required for definitive diagnosis.

The diagnosis of *C. sinensis* infection was established on the basis of microscopic identification of eggs in stool or detection of adult parasites in bile or pathological specimens [28, 29]. Simultaneously, we evaluated the co-infection status of hepatitis B virus (HBV) and hepatitis C virus (HCV) among all study participants. We collected serological test results for HBV and HCV and adjusted the analysis accordingly when assessing the correlation with *C. sinensis* infection. All patients were infected with HBV but not HCV. The diagnosis of HBV infection was confirmed by the detection of hepatitis B surface antigen (HBsAg) in preoperative serum samples, with a positive qualitative result indicating active infection [30]. The diagnosis of HCV infection was based on the detection of anti-HCV antibodies in preoperative serum samples [31].

### Bioinformatic analysis

The study utilized GEPIA (http://gepia.cancer-pku.cn/index.html) to explore the expression of CD24 in HCC, UALCAN (http://ualcan.path.uab.edu/) to analyze the relationship between CD24 mRNA expression in HCC and various pathological parameters, and TIMER2.0 (http://timer.cistrome.org/) to assess the correlation between CD24 expression, immune cell infiltration, and immune checkpoints. Finally, we employed the Kaplan–Meier plotter database (http://kmplot.com) to evaluate the prognostic significance of CD24 regarding OS, progression-free survival (PFS), recurrence free survival (RFS), and disease-specific survival (DSS). The analysis included hazard ratios (HR) with 95% confidence intervals (95% CI) and log-rank *P*-values. Potential transcription factors binding to the CD24 promoter region were predicted using the PROMO (https://alggen.lsi.upc.es/cgi-bin/promo_v3/promo/promoinit.cgi?dirDB=TF_8.3) and Harmonizome (https://maayanlab.cloud/Harmonizome/) databases.

### Preparation CsESPs

*Pseudorasbora parvas*, recognized as the second intermediate host for *C. sinensis*, was sourced from Hengzhou, Guangxi, exhibiting natural infection with metacercariae. The fish specimens underwent digestion with an artificial gastric solution composed of 0.5% pepsin, 1% hydrochloric acid, and 0.9% sodium chloride NaCl, maintained at 37 °C overnight. Male Sprague–Dawley rats were procured from the Guangxi Medical University Laboratory Animal Center and were cared for in compliance with the National Institutes of Health’s standards for animal welfare and ethical treatment (ethics approval no.: 202410011). Metacercariae were extracted using a stereomicroscope, and a total of 150 metacercariae were administered intragastrically to each rat. Following an 8-week infection period, the rats were euthanized post-anesthesia, allowing for the collection of adult worms from the bile ducts, which were subsequently cultured in phosphate-buffered saline (PBS), maintained at 37 °C in an atmosphere containing 5% CO_2_. The culture medium was collected after a duration of 6 h, followed by centrifugation at 12,000 × g for 10 min at 4 °C. The resulting supernatant, designated as CsESPs, was sterilized through a 0.22 μm filter and preserved at −80 °C. The concentrations of CsESPs were quantified utilizing the bicinchoninic acid assay.

### Cell culture

Human HCC cell lines Huh7 and Hep3B were obtained from the Shanghai Institute of Biochemistry and Cell Biology (Shanghai, China) and validated through STR identification. These cell lines were maintained in Dulbecco’s Modified Eagle’s Medium enriched with 10% fetal bovine serum and 1% penicillin–streptomycin.

### Cell transfection

Short interfering RNAs (siRNAs) targeting CD24 were synthesized by GenePharma (Shanghai, China) and conducted as previously described [32]. For the siRNA sequences, please refer to Supplementary Table 1 for details. The silencing effect on mRNA expressions were examined by qPCR analysis.

### Detection of cell viability and colony formation

Cell viability was evaluated utilizing the Cell Counting Kit-8 (CCK-8) assay. Huh7 and Hep3B cell lines were exposed to 100 µg/mL concentrations of CsESPs for a duration of 48 h. Subsequently, CCK-8 was combined with the culture medium at a ratio of 1:10, applied to each well, and incubated for 1.5 h, in accordance with previously established protocols, and optical density (OD) was assessed at a wavelength of 450 nm [33]. Cell viability was calculated as follows: cell viability = (OD _treatment_ − OD _blank_)/ (OD _control_ − OD _blank_), where OD _treatment_, OD _control_, and OD _blank_ represent the absorbance values of the treatment group, untreated control group, and blank wells, respectively. The colony formation assay was conducted as previously outlined [34], with 1000 cells being plated in 12-well plates and subjected to treatment with the specified drugs over a period of 10 days. After performing crystal violet staining, photographic documentation of the colonies was obtained.

### Apoptosis assay

To evaluate cell apoptosis, we utilized the Annexin V-APC/PI Apoptosis Kit (Lianke). In summary, HCC cells were collected via accutase treatment. Following this, the cells were resuspended in binding buffer and subsequently stained with 5 µl of Annexin V- allophycocyanin (APC) and Propidium Iodide (PI) for a duration of 5 min. The analysis of apoptosis was conducted using a CytoFLEX Flow Cytometer (Beckman-Coulter). Our pre-gating strategy includes: (1) a scatter plot gating by forward scatter (FSC) & side scatter (SSC) to exclude non-cellular events (e.g., debris); (2) another scatter plot gating by FSC-height&FSC-area to exclude cell dimer events. Scatter plots for APC and PI are set to distinguish live cells (Annexin V-APC^−^ & PI^−^), early apoptotic cells (Annexin V-APC^+^ & PI^−^), late apoptotic cells (Annexin V-APC^+^ & PI^+^), and necrotic cells (Annexin V-APC^−^ & PI^+^). The percentage of apoptotic cells is the sum of early apoptotic cells and late apoptotic cells.

### Western blotting (WB) assay and immunofluorescence

WB was performed as previously described [35]. Primary antibodies E2F1 (sc-251) were obtained from Santa Cruz Biotechnology (USA); CD24 (67627–1-Ig), Bcl-2-associated X protein (BAX) (50599–2-Ig), β-actin (66009–1-Ig), and *α*-Tubulin (66031–1-Ig) from Proteintech (China). Immunofluorescence was performed as described previously [36]. Observational data and imaging were captured utilizing a confocal microscope (Zeiss LSM 800).

### Real-time quantitative polymerase chain reaction (qPCR)

RNA extraction was performed utilizing TRIzol reagent in accordance with the guidelines provided by the manufacturer. To assess mRNA expression levels, qPCR was conducted employing the 2 × Universal Blue SYBR Green qPCR Master Mix (Servicebio), adhering strictly to the manufacturer’s protocol. Glyceraldehyde-3-phosphate dehydrogenase (GAPDH) served as an internal control for normalization. All primers utilized for qPCR were procured from Sangon Biotechnology Ltd., and the specific sequences of these primers are detailed in Supplementary Table 2.

### Dual-Luciferase Reporter Assay

The wild-type and mutant luciferase plasmids of the CD24 promoter were constructed by Hunan Fenghui Biotechnology Co., Ltd. (China). Similarly, the vector and overexpression plasmids of E2F1 were constructed by Wuhan MiaoLing Biotechnology Co., Ltd. (China). The binding sites of E2F1 on the CD24 promoter were predicted using the JASPAR database (https://jaspar.elixir.no/). HEK-293 T cells were initially resuspended and subsequently plated into 6-well culture plates. The CD24 promoter luciferase plasmids were co-transfected into the HEK-293 T cells alongside either a control vector or plasmids designed for E2F1 overexpression. Following a 48-h incubation period, cell lysates were harvested, and the activities of firefly and renilla luciferase were quantified utilizing the Dual-Luciferase Reporter Assay Kits (Promega).

### Chromatin immunoprecipitation (ChIP) assay

ChIP was executed in accordance with the guidelines provided by the manufacturer (Beyotime Biotechnology). In summary, cells were harvested and subjected to crosslinking using 1% formaldehyde. Subsequently, the DNA underwent fragmentation through sonication. Prewashing was conducted with agarose beads, and then the mixture was incubated with 5 μg of E2F1 (Santa Cruz, sc-251) in the culture medium. Ultimately, DNA fragments were isolated utilizing magnetic beads, followed by quantification through qPCR.

### Isolation of peripheral blood mononuclear cells (PBMCs)

PBMCs were extracted from EDTA-anticoagulated peripheral blood obtained from healthy volunteers, which was approved by the medical ethics committee of Guangxi Medical University (ethics approval no.: KY20250251). The whole blood sample was diluted with an equivalent volume of PBS and carefully layered over an equal volume of lymphocyte separation medium. PBMCs were then isolated by gradient centrifugation. Following centrifugation at 800 g for 25 min, the mononuclear cell layer was harvested and washed three times with PBS.

### Co-culture of HCC cells with PBMCs

PBMCs and HCC cells were co-cultured using a non-contact co-culture transwell system (JETBIOFIL, China). HCC cells were maintained in the upper chamber, whereas PBMCs were grown in the lower chamber, using RPMI-1640 medium enriched with 10% fetal bovine serum and 1% penicillin–streptomycin. After 48 h of co-incubation, PBMCs were harvested for further analyses.

### Statistical analysis

Data analysis was conducted utilizing SPSS software (version 22.0), with results expressed as the mean ± standard error of the mean (SEM). Statistical visualizations were created employing GraphPad Prism 8 software. Group comparisons were carried out using either the independent samples *t*-test or the Mann–Whitney *U*-test, depending on the data distribution. Alternatively, one-way analysis of variance (ANOVA) was employed, followed by multiple comparisons using either the least significant difference (LSD) test or the Games–Howell correction. A *P*-value of less than 0.05 was considered statistically significant.

## Results

### CD24 expression is up-regulated in HCC

**Fig 1.**
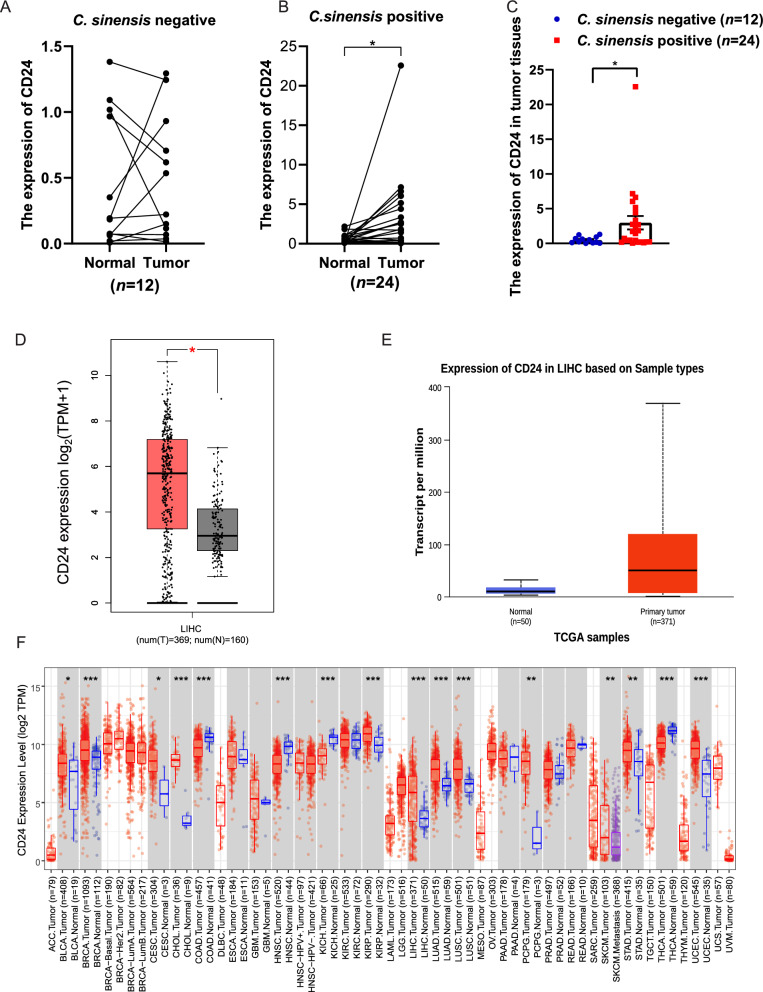
The expression of CD24 in *C. sinensis*-associated HCC. **A–B**. qPCR analysis of CD24 expression in 12 pairs of tumor and adjacent normal tissues from *C. sinensis*-negative patients with HCC (**A**) and in 24 pairs of tumor and adjacent normal tissues from *C. sinensis*-positive patients with HCC (**B**). ^*^P < 0.05 compared with Normal. **C**. Comparisons of CD24 expression between *C. sinensis*-positive and *C. sinensis*-negative tumor tissues. ^*^P < 0.05 compared with *C. sinensis*-negative tumor tissues. **D**. Elevated CD24 expression in LIHC (liver hepatocellular carcinoma) compared with normal tissues, as shown in the GEPIA database. **E**. Elevated CD24 expression in LIHC compared with normal tissues, as shown in the UALCAN database. **F**. CD24 expression across various cancer types, analyzed using the TIMER2.0 database. ^*^P < 0.05, ^**^P < 0.01, ^***^P < 0.001 compared with Normal

Using our in-house cohort, we evaluated CD24 mRNA expression in HCC tumor and paired adjacent nontumor tissues. qPCR analysis revealed significant upregulation of CD24 in *C. sinensis*-infected HCC tumor tissues compared with adjacent tissues (Fig. 1A–B). Further analysis revealed that the expression of CD24 was much higher in HCC tissues positive for *C. sinensis* than in HCC tissues negative for *C. sinensis* (Fig. 1C). To validate these findings, we conducted comprehensive bioinformatic analyses using multiple independent datasets (GEPIA, UALCAN, and TIMER2.0). Consistent with our experimental results, database analyses confirmed that CD24 expression was significantly increased in HCC tissues when compared with those of normal liver tissues. (Fig. 1D–E). Furthermore, pan-cancer analysis through TIMER2.0 demonstrated significantly higher CD24 expression in multiple malignancies relative to their corresponding normal tissues (Fig. 1F). These consistent findings across experimental and bioinformatic approaches suggest that CD24 upregulation is particularly prominent in *C. sinensis*-associated HCC and could be crucial in the development of liver cancer and the advancement of tumors.

### CD24 expression and different HCC clinicopathological parameters

**Fig 2.**
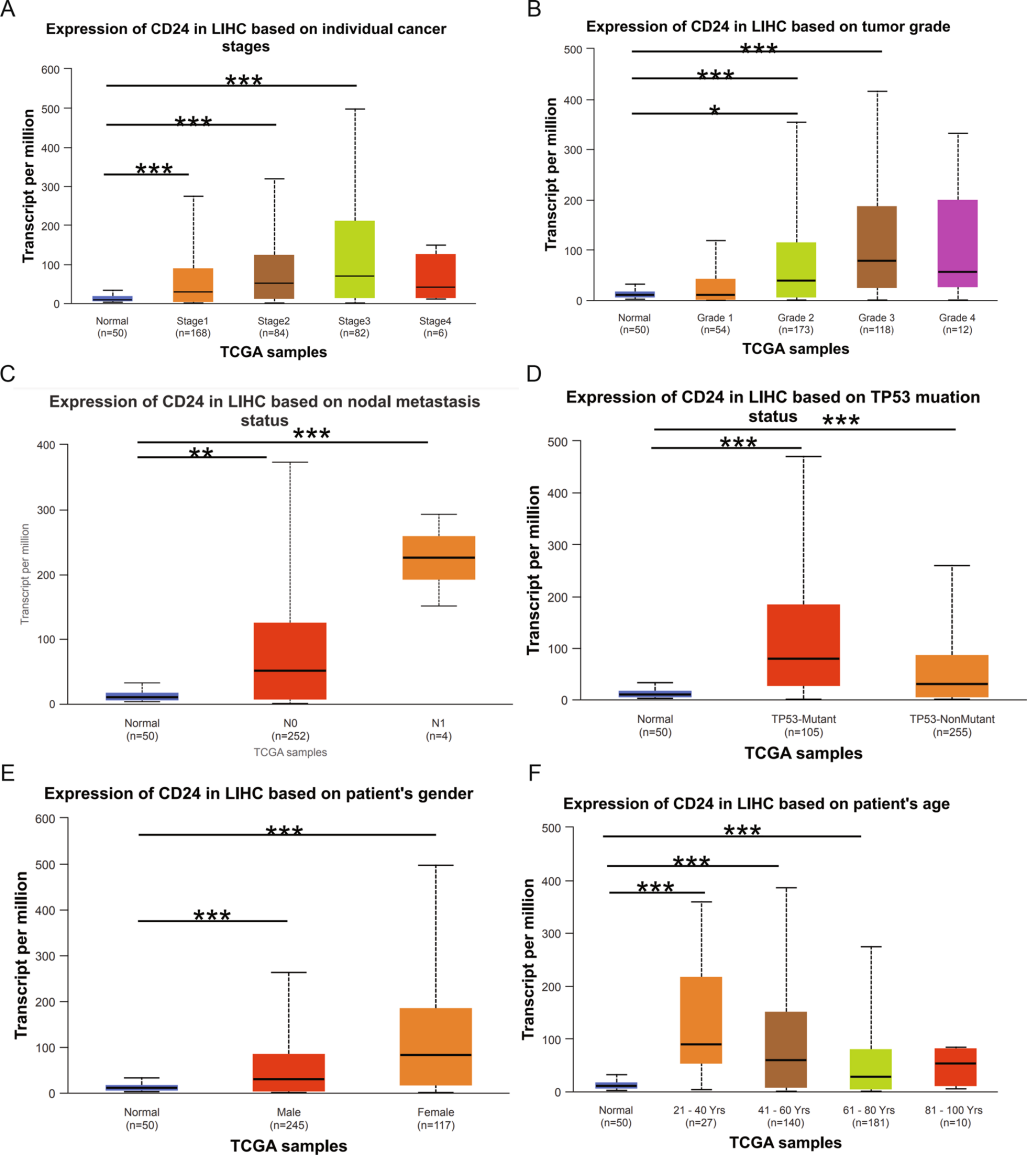
Box plots of CD24 expression across patient groups stratified by clinical parameters using the UALCAN database.** A**. CD24 expression across individual cancer stages. **B**. CD24 expression based on tumor grade. **C**. CD24 expression according to nodal metastasis status. **D**. CD24 expression stratified by TP53 mutation status. **E**. CD24 expression based on gender. **F**. CD24 expression across different age groups.^*^P < 0.05, ^**^P < 0.01, ^***^P < 0.001 compared with Normal

Given the marked elevation of CD24 expression in HCC, we systematically evaluated its clinical relevance using the UALCAN database. Our analysis revealed several key findings. First, CD24 expression showed progressive upregulation across advancing tumor stages (stages 1–3) compared with normal liver tissue (Fig. 2A). This stage-dependent pattern was paralleled by Edmondson’s pathological grading, with significantly higher CD24 levels in grades 1–3 HCC (Fig. 2B). Notably, CD24 expression was particularly elevated in cases with nodal metastasis (Fig. 2C), suggesting a potential role in disease dissemination. Further stratification analysis demonstrated that CD24 upregulation occurred independently of TP53 mutation status (Fig. 2D), patient gender (Fig. 2E), and aging (21–80 years) (Fig. 2F). In all subgroups examined, CD24 expression remained consistently higher in tumor tissue than in normal liver tissues. These comprehensive analyses establish CD24 as a robust biomarker consistently associated with HCC progression across multiple clinical parameters.

### High CD24 expression is associated with poor prognosis in HCC

**Fig 3.**
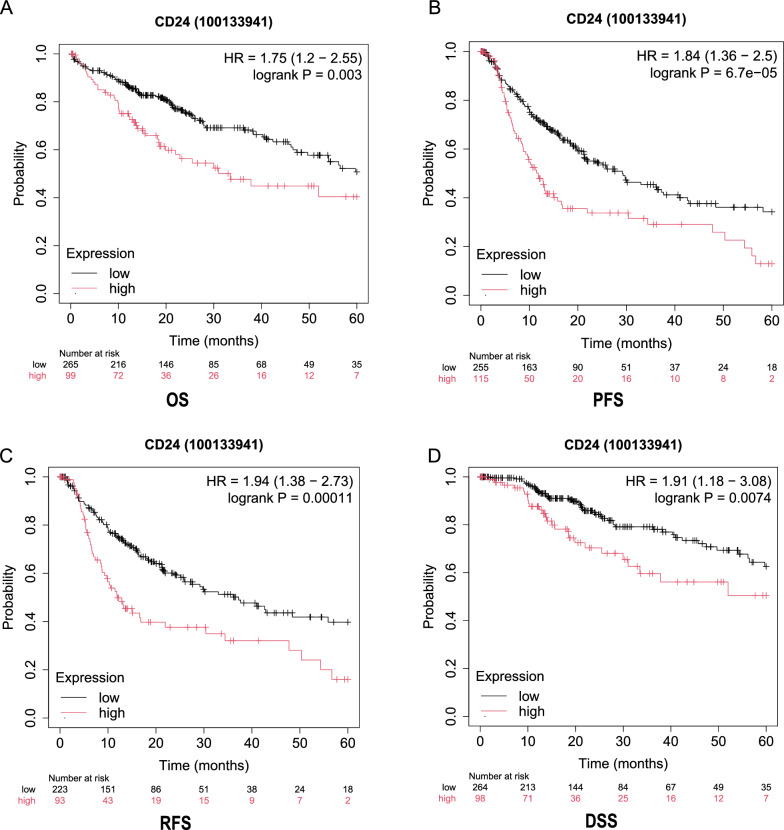
Kaplan–Meier survival curves assessing the prognostic value of CD24. **A**. Overall survival (OS). **B**. Progression-free survival (PFS). **C**. Recurrence-free survival (RFS). **D**. Disease-specific survival (DSS)

To assess the prognostic value of CD24 in HCC, we performed survival analyses using the Kaplan–Meier plotter database. Patients were dichotomized into high- and low-expression groups on the basis of median CD24 expression level. The results demonstrated that the CD24-high patients with HCC exhibited significantly worse OS, PFS, RFS, and DSS compared with those with low CD24 expression (Fig. 3). Furthermore, we assessed the prognostic significance of CD24 in relation to diverse clinicopathological characteristics by utilizing the Kaplan–Meier database. Especially in those with vascular invasion, higher CD24 expression was substantially linked to worse OS and PFS (Supplementary Table 3). Collectively, these findings indicate that CD24 serves as a significant prognostic marker in HCC, highlighting its potential clinical relevance.

### Correlation analysis of CD24 expression with immune cell infiltration

**Fig 4.**
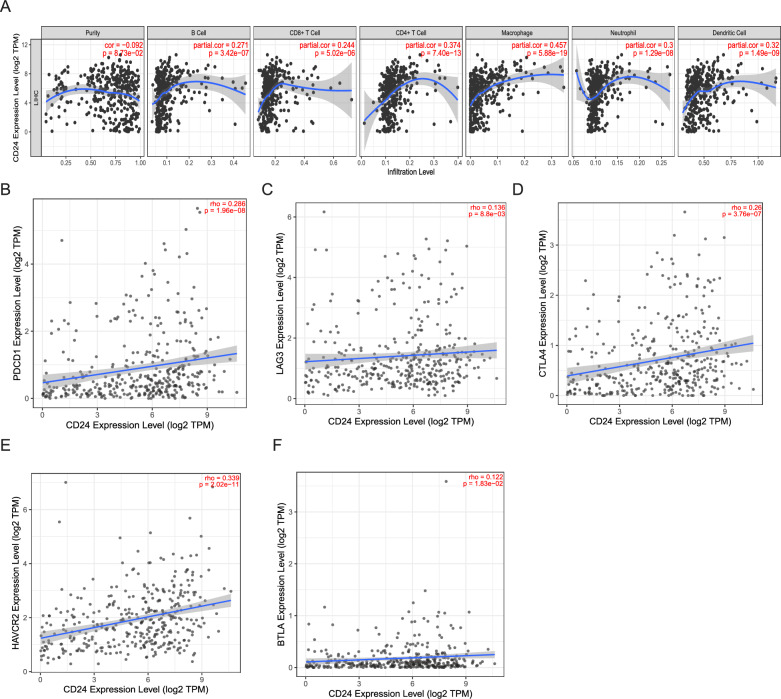
Relationship between CD24 and immune cells. **A**. CD24 shows a significant association with tumor purity and positively correlates with the infiltration of various immune cells, as analyzed using the TIMER 2.0 database. **B–D**. Using the TIMER2.0 database, scatterplots illustrating the correlations between CD24 expression and immune checkpoint markers in LIHC, including programmed cell death protein-1 (PDCD1, panel **B**), lymphocyte-activation gene 3 (LAG3, panel **C**), cytotoxic T lymphocyte-associated antigen-4 (CTLA-4, panel **D**), hepatitis A virus cell receptor 2 (HAVCR2, panel **E**), and B and T lymphocyte attenuator (BTLA, panel **F**)

Given the complexity of the liver immune microenvironment, we systematically evaluated the correlation between CD24 expression and the tumor immune microenvironment characteristics using the TIMER2.0 database. Our comprehensive analysis revealed several key findings. CD24 expression exhibited notable positive correlations with the infiltration levels of six predominant immune cell types, namely B cells, CD8^+^ T cells, CD4^+^ T cells, macrophages, neutrophils, and dendritic cells (Fig. 4A). To further investigate the association between CD24 and immune responses, we analyzed the relationship between CD24 expression and a range of immune characteristics in HCC. These included evaluations of B cells, T cells, CD8^+^ T cells, monocytes, tumor-associated macrophages (TAMs), M1/M2 macrophages, neutrophils, natural killer cells, and dendritic cells. Following adjustments for tumor purity, our analysis confirmed a significant correlation between CD24 expression and most established markers representative of these immune cell types (Supplementary Table 4). Additionally, we identified a positive correlation between CD24 expression levels and various immune checkpoints, including CTLA-4, LAG3, PDCD1, HAVCR2, and BTLA (Fig. 4B-F). Collectively, these results indicate that CD24 may serve a crucial function in the regulation of immune infiltration in HCC, thereby underscoring its potential as a target for therapeutic intervention.

### CsESPs promote proliferation and inhibit apoptosis in HCC cells

**Fig 5.**
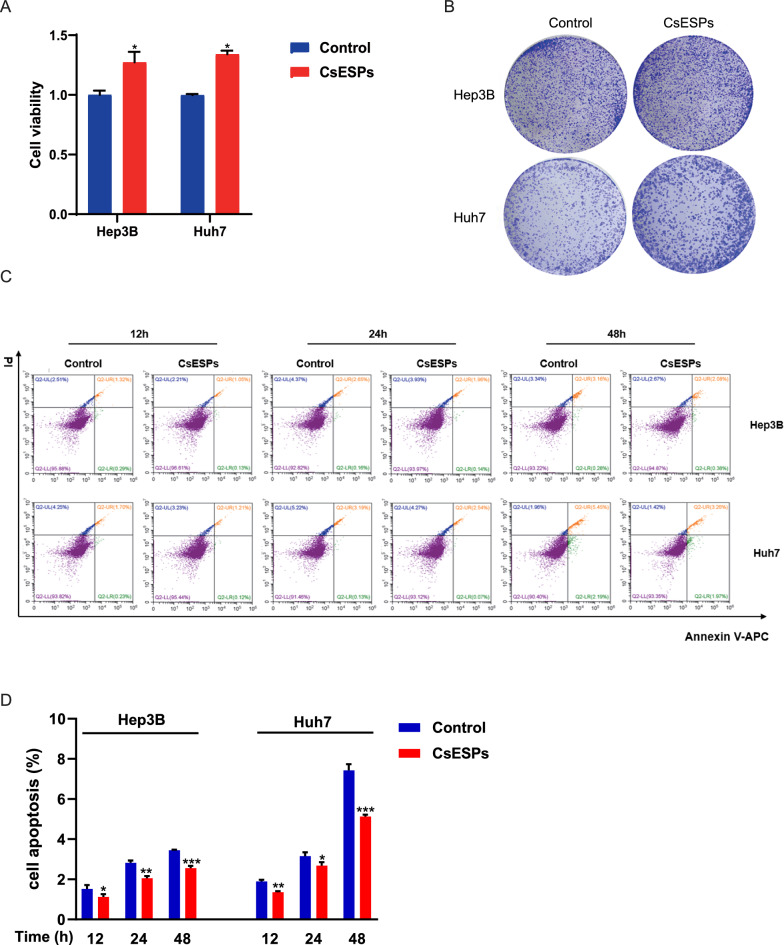
Exposure to CsESPs promotes cell proliferation and inhibits apoptosis in HCC cells. **A**. Cell viability was assessed using the CCK8 assay in HCC cells treated or untreated with CsESPs (100 μg/mL) for 48 h. **B**. Cell proliferation was evaluated by colony formation assay in HCC cells treated or untreated with CsESPs (100 μg/mL). **C–D**. Apoptosis was measured by flow cytometry in control and CsESPs-treated HCC cells at different timepoints (12 h, 24 h, and 48 h) and summarized in panel D. ^*^P < 0.05, ^*^^*^P < 0.01, ^***^P < 0.001 compared with Control

To investigate the effects of CsESPs on HCC cells, we treated Hep3B and Huh7 cells with 100 μg/mL CsESPs. CsESPs treatment significantly enhanced the viability of HCC cells after 48 h of exposure and clonogenic proliferation compared with the control group (Fig. 5A-B). Furthermore, flow cytometry analysis demonstrated that CsESPs markedly reduced basal level of apoptosis in HCC cells in a time-dependent manner (Fig. 5C-D). Collectively, these results indicate that CsESPs promote HCC cell proliferation and suppress apoptosis, suggesting a potential role in HCC progression.

### CsESPs may up-regulate CD24 expression through transcription factor E2F1

**Fig 6.**
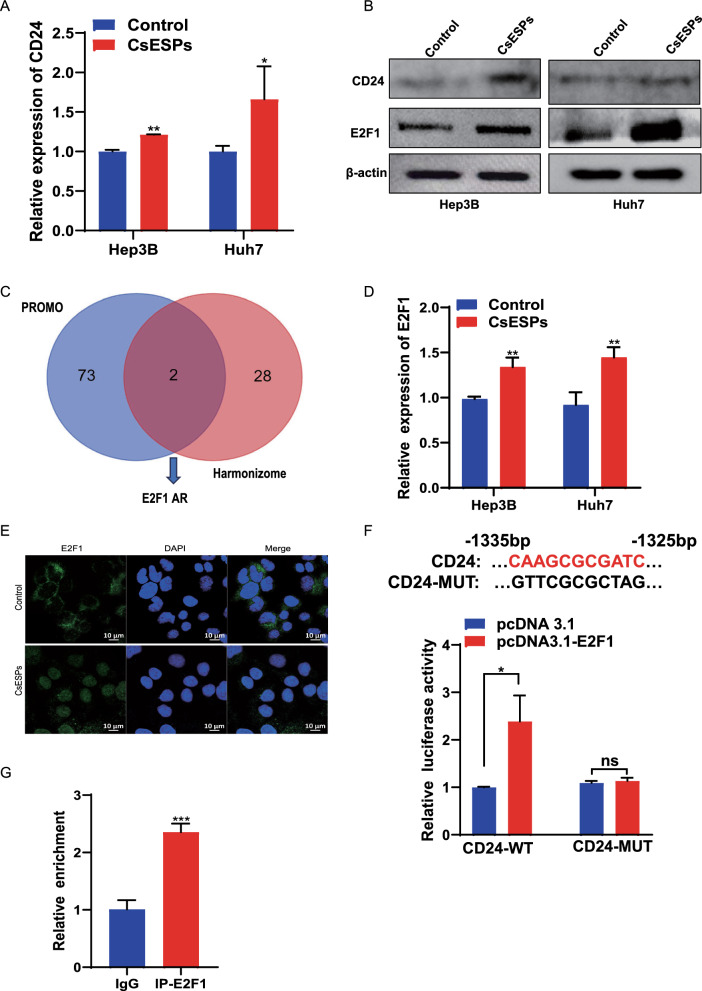
CsESPs upregulates CD24 expression by enhancing E2F1 expression. **A**. CD24 expression was detected by qPCR in control and CsESPs-treated HCC cells. ^*^P  < 0.05, ^**^P < 0.01 compared with Control. **B**. Protein expression levels of CD24 and E2F1 were analyzed by WB in HCC cells treated or untreated with CsESPs. **C**. Schematic representation of overlapping transcription factors of CD24 predicted by PROMO and Harmonizome. **D**. E2F1 expression was detected by qPCR in control and CsESPs-treated HCC cells. ^*^P < 0.05, ^**^P < 0.01 compared with Control. **E**. Subcellular localization of E2F1 was determined by immunofluorescence (scale bar = 10 μm). **F**. Potential binding sites of E2F1 on the CD24 promoter sequence and corresponding mutations. CD24 promoter luciferase activity was assessed by dual-luciferase assays. ns: no significance, ^*^P < 0.05 compared with pcDNA3.1. **G**. ChIP-qPCR demonstrated that E2F1 binds to the promoter regions of CD24 in Huh7 cells. ^*^P < 0.05, ^**^P < 0.01, ^***^P < 0.001 compared with immunoglobulin (IgG).

Consistent with the *C.sinensis*-positive tumor tissue  changes (Fig. 1B), CD24 was significantly upregulated in CsESPs-treated HCC cells (Fig. 6A and 6B). To investigate the mechanism underlying CD24 upregulation by CsESPs, we predicted potential transcription factors binding to the CD24 promoter region using both PROMO and Harmonizome databases. By intersecting the results from both databases, we identified E2F1 and AR as candidate transcription factors (Fig. 6C). Subsequent correlation analysis using the GEPIA database revealed that E2F1 exhibited a positive correlation with CD24, in contrast, AR showed a negative correlation with CD24 (Supplementary figure 1). Further WB and qPCR experiments confirmed that E2F1 was up-regulated in CsESPs-treated HCC cells (Fig. 6B and D). Immunofluorescence assays also indicated that E2F1 translocated from the cytoplasm to the nucleus following CsESPs treatment (Fig. 6E).

To validate the binding of E2F1 to the CD24 promoter, we used the JASPAR database to predict potential binding sites. The region spanning 1325–1335 bp scored highly, suggesting strong binding affinity. We subsequently constructed E2F1 overexpression vectors and CD24 promoter vectors with either wild-type or mutated binding sites. Dual-luciferase reporter assays revealed that E2F1 enhanced luciferase activity conjugating wild-type CD24 promoter sequence, but not in those with mutant sequence (Fig. 6F). More importantly, ChIP-qPCR confirmed that E2F1 binds to the CD24 promoter region (Fig. 6G). Collectively, these findings demonstrate that CsESPs upregulate CD24 expression through transcriptional regulation by E2F1.

### CD24 silencing abolishes CsESPs-induced cell proliferation and apoptosis reduction

**Fig 7.**
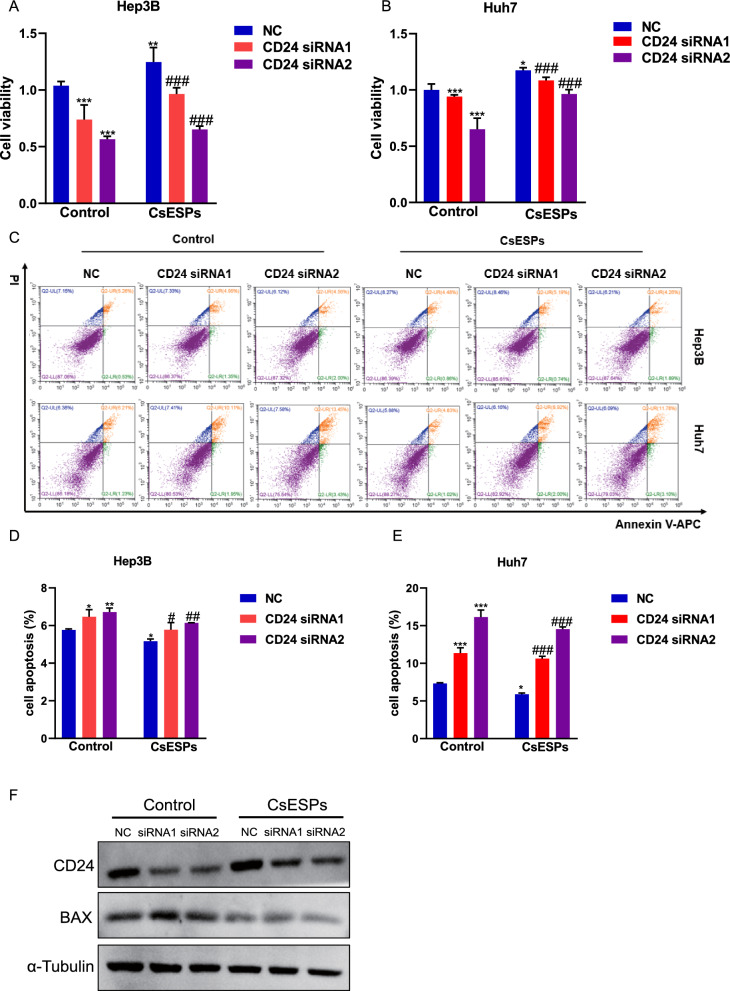
CD24 promotes cell viability and inhibits apoptosis in HCC cells under CsESPs exposure. **A–B**. Cell viability was assessed by CCK-8 assay in HCC cells transfected with si-CD24 or negative control (NC) and treated or untreated with CsESPs. **C–E**. Apoptosis was measured by flow cytometry in HCC cells transfected with si-CD24 or NC and treated or untreated with CsESPs. **F**. Protein expression levels of CD24 and BAX were analyzed by western blot in HCC cells treated or untreated with CsESPs. ^*^P < 0.05, ^**^P < 0.01, ^***^P < 0.001 compared with control NC. ^#^*P* < 0.05, ^##^*P* < 0.01, ^###^*P* < 0.001 compared with CsESPs NC

To investigate the essential role of CD24 in CsESPs-treated HCC cells, we performed knockdown of CD24 using specific siRNA (Supplementary figure 2). As expected, CD24 silencing significantly reduced the viability of CsESPs-treated HCC cells (Fig. 7A–B). Flow cytometry analysis further demonstrated that CD24 knockdown markedly increased apoptosis in these cells (Fig. 7C–E). WB assays revealed elevated expression of the pro-apoptotic protein BAX in CD24-knockdown cells (Fig. 7F). Together, these results confirm that CD24 promotes cell proliferation and inhibits apoptosis in CsESPs-treated HCC cells.

### Relationship between CD24 and immune checkpoints in CsESPs-treated HCC cells

**Fig 8.**
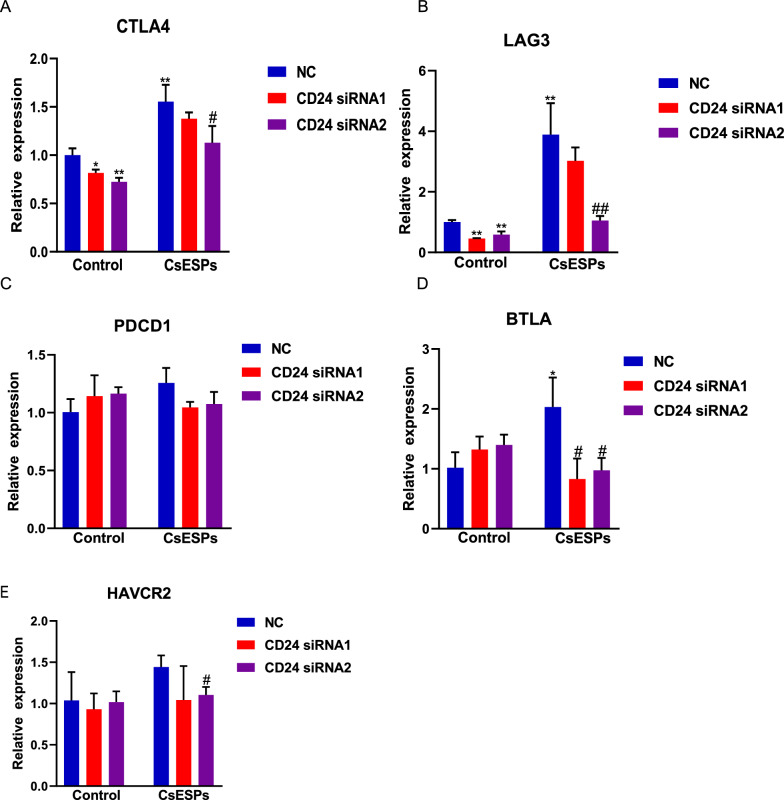
Relationship between CD24 and immune checkpoints in HCC cells under CsESP exposure. **A-E**. In PBMCs co-cultured with HCC cells transfected with si-CD24 or NC and treated or untreated with CsESPs, qPCR analyses determined the expression levels of different immune checkpoints, including CTLA4: cytotoxic T lymphocyte-associated antigen-4 (CTLA4, panel **A**), lymphocyte-activation gene 3 (LAG3, panel **B**), programmed cell death protein-1 (PDCD1, panel **C**), B and T lymphocyte attenuator (BTLA, panel **D**) and hepatitis A virus cell receptor (HAVCR2, panel **E**). ^*^P < 0.05, ^**^P < 0.01, ^***^P < 0.001 compared with control NC. ^#^*P* < 0.05, ^##^*P* < 0.01, ^###^*P* < 0.001 compared with CsESPs NC

To further investigate whether CD24 up-regulation in CsESPs-treated HCC cells affect multiple immune checkpoint molecules, we established an in vitro co-culture system of HCC cells and PBMCs. qPCR analyses showed that CD24 knockdown significantly attenuated CsESPs-induced upregulation of CTLA-4 and LAG-3 (Fig. 8A–B). However, CD24 knockdown failed to alter the levels of other genes associated with immune checkpoints, such as PDCD1, HAVCR2, and BTLA (Fig. 8C–E). These results suggest that CD24 may selectively regulate specific immune checkpoint pathways in CsESP-treated HCC cells.

## Discussion

This study demonstrated that CD24 is significantly upregulated in HCC, particularly in cases associated with *C. sinensis*. Analysis from multiple databases indicates that the level of CD24 expression correlates with clinicopathological stages and prognosis in patients with HCC. Mechanistically, CsESPs can promote proliferation and inhibit apoptosis of HCC cells through E2F1-mediated transcriptional regulation of CD24. Moreover, CsESPs up-regulated the immune checkpoint molecules CTLA-4 and LAG-3 in the co-cultured PBMCs, an effect that could be reversed by knockdown of CD24.

Consistent with the up-regulation of CD24 in multiple malignancies [37], our preliminary single-cell sequencing data revealed elevated CD24 expression in *C. sinensis*-associated HCC. We further validated its up-regulation in both in-house cohorts and online datasets. More importantly, the Kaplan–Meier and Cox regression analyses demonstrated that aberrant CD24 expression has the potential to act as a prognostic biomarker for HCC, consistent with prior research [27]. Furthermore, CD24 exhibits a strong association with immune checkpoint genes, suggesting its potential role in modulating tumor immune infiltration. Given its differential expression patterns and immunomodulatory implications, we hypothesize that CD24 contributes to tumorigenesis and progression in a context-dependent manner, possibly influencing immunotherapy responsiveness.

Clonorchiasis, caused by the *C. sinensis*, is recognized as one of the most clinically overlooked tropical diseases in East Asia [38]. This persistent *C. sinensis* infection is recognized as a risk factor for cholangiocarcinoma development [12, 39]. Recent epidemiological evidence also associates *C. sinensis* infection with poorer clinical outcomes in patients with HCC [8, 23, 24]. Notably, the parasite enhances malignant transformation through induction of cancer stem cell-like properties [8]. The molecular mechanisms underlying this oncogenic effect involve parasite-derived factors such as the calcium-binding protein Cs16, which drives hepatic inflammation by reprogramming metabolic pathways in innate immune cells [40]. Similarly, CsESPs contribute to infection-associated pathology. For instance, the CsESPs component Csseverin exhibits potent anti-apoptotic effects in human HCC cell lines [41]. In alignment with these findings, our current study demonstrates that CsESPs promote HCC cell proliferation while suppressing apoptosis. Importantly, we observed that CD24-upregulation may play an essential role in CsESPs-induced cell proliferation enhancement and apoptosis reduction in HCC cells.

E2F1, a pivotal transcription factor regulating diverse cellular processes such as proliferation, differentiation, migration, and metabolism [42], exhibits dual oncogenic and tumor-suppressive roles in cancer progression [43, 44]. In this study, we found that CsESPs treatment significantly up-regulates E2F1 expression in HCC cells and induces its nuclear translocation. Mechanistic analysis revealed that E2F1 can directly binds to the CD24 promoter region, thereby enhancing its transcriptional activity. These findings suggest that CsESPs may upregulate CD24 expression by increasing E2F1 levels.

Beyond E2F1, other pathways have been implicated in CD24 regulation. For example, the RNA-editing enzyme ADAR promotes HCC progression by enhancing CD24 expression through interaction with SNRPD3 and RNPS1, which inhibit STAU1-mediated mRNA degradation [45]. Additionally, the Hippo-YAP1-SOX4 axis has been identified as a critical regulatory pathway for YAP1-mediated CD24 overexpression in HCC cells, with CD24 suppression markedly inhibiting YAP1-driven HCC progression [46].

Previous studies have shown that Csseverin, a component of CsESPs, protects human HCC cells from apoptosis by inactivating membrane Ca^2+^ channels, potentially contributing to HCC progression in the context of *C. sinensis* infection [47]. Another key component, growth factor CsGRN, induces epithelial–mesenchymal transition (EMT) and promotes HCC metastasis by activating the PI3K/AKT and ERK pathways [18]. CsGRN also facilitates the malignant transformation of normal liver cells by modulating EGFR-mediated RAS/MAPK/ERK and PI3K/AKT signaling pathways [19]. Furthermore, overexpression of secreted phospholipase A_2_ (CsGIIIsPLA_2_) from *C. sinensis* inhibits HCC cell proliferation while enhancing migration and EMT through AKT activation-mediated signaling [48].

Our experimental findings underscore the critical role of CD24 in CsESPs-mediated HCC pathogenesis. CD24 knockdown in CsESPs-treated HCC cells significantly reduced cell viability, increased apoptosis, and elevated BAX expression. Collectively, these results suggest that CsESPs upregulate CD24 expression via transcriptional regulation of E2F1, thereby promoting proliferation and inhibiting apoptosis in liver cancer cells.

More importantly, our findings demonstrate that CD24 expression positively correlates with immune checkpoints, including LAG-3 and CTLA-4. Importantly, CsESPs-induced upregulation of these checkpoints was attenuated by CD24 knockdown, suggesting CD24’s pivotal role in mediating immune evasion in CsESPs-treated HCC cells. As critical immune checkpoint molecules, CTLA-4 and LAG-3 suppress T cell activation through distinct mechanisms. CTLA-4 competitively binds to B7 molecules with higher affinity than CD28, thereby inhibiting co-stimulatory signals, while LAG-3 negatively regulates T cell responses via MHC class II interactions. Co-expression of these checkpoints on exhausted T cells drives progressive functional impairment, characterized by diminished cytokine production and cytotoxic activity. Notably, recent evidence demonstrates that dual CTLA-4/LAG-3 blockade synergistically restores T cell function, highlighting their cooperative role in maintaining T cell exhaustion [49]. T cell exhaustion, a dysfunctional state marked by TOX-driven transcriptional reprogramming and sustained expression of inhibitory receptors (e.g., LAG-3, CTLA-4), is a hallmark of chronic viral infections and tumor microenvironments [50]. This exhaustion leads to CD8^+^ T cell proliferation failure and loss of effector capacity, ultimately compromising antitumor immunity. These results position CD24 as a potential regulator of T cell exhaustion and a therapeutic target for restoring antitumor immunity.

While this study provides novel insights into CD24’s role in *C. sinensis*-associated HCC pathogenesis, several limitations should be acknowledged. First, the study lacks in vivo validation, as findings derived from the in vitro experiments may not fully reflect the complexity of HCC progression. Clarification of HCC tumor microenvironment through single-cell RNA sequencing (scRNA-seq) and spatial transcriptomic sequencing (ST-seq) may shed light on this question. Second, the relatively small sample size in the in-house HCC cohort may limit the generalizability of the results. Particularly, it is yet unknown whether *C. sinensis* infection affect the treatment response and long-term prognosis of immunotherapy, which is increasingly adopted as first-line HCC treatment. Third, CsESPs may not fully represent the oncogenic ability of *C. sinensis.* The released noncoding RNAs and metabolites by extracellular vesicle by parasite are also important oncogenic factors. Addressing these limitations in future studies will be essential to dissect the oncogenic effects of *C. sinensis*-associated HCC and the potential application of CD24 as a predictive biomarker and potential therapeutic target.

## Conclusions

This study comprehensively establishes CD24 as a clinically significant biomarker in HCC, with particularly pronounced overexpression in *C. sinensis*-associated cases. Integrated multi-database analyses revealed that CD24 expression levels not only correlate with advanced clinicopathological stages, but also act as a standalone prognostic marker in patients with HCC. Mechanistically, CsESPs upregulates E2F1, which subsequently activates CD24 transcription, ultimately promoting tumor cell proliferation while suppressing apoptosis. Furthermore, CsESPs treatment increases the levels of the immune checkpoint molecules CTLA-4 and LAG-3 in co-cultured PBMCs—an effect that was reversible by CD24 knockdown. These results reveal the essential roles of CD24 in both tumor-intrinsic growth pathways within HCC cells and immune evasion mechanisms in microenvironment in *C. sinensis*-associated HCC.

## Supplementary Information

Additional file 1.

## Data Availability

Data supporting the main conclusions of this study are included in the manuscript.

## Abbreviations

CD24: Cluster of differentiation 24
*C. sinensis*: *Clonorchis sinensis*
CsESPs: *Clonorchis sinensis* excretory–secretory products
DSS: Disease-specific survival
OS: Overall survival
PBS: Phosphate-buffered saline
PFS: Progression-free survival
RFS: Recurrence-free survival
