# Supplementary material for: Clonorchis sinensis-driven hepatocarcinogenesis via E2F1-CD24 transcriptional axis: mechanistic and therapeutic implications
Source: Parasit Vectors. 2025 Aug 19;18:353. doi: 10.1186/s13071-025-06979-6 (PMC12366411; doi:10.1186/s13071-025-06979-6)
Supplement: Supplementary file 1 — Additional file 1. [file 13071_2025_6979_MOESM1_ESM.docx]

**Supplemental Tables**

Supplementary Table 1. RNA oligonucleotide sequences of siRNAs.

| RNA oligos | Sequence |
| --- | --- |
| CD24 Negative Control (NC) | ACGUGACACGUUCGGAGAATT |
| CD24 siRNA1 | AUUAGUUGGAUUUGGGGCCTT |
| CD24 siRNA2 | AGUGAGACCACGAAGAGACTT |
| CD24 siRNA3 | AAAUCUGCGUGGGUAGGAGTT |

Supplementary Table 2. The primers for qPCR.

| Gene | | Sequence（5’-3’） |
| --- | --- | --- |
| GAPDH | Forward | AGCAAGTGCAACTACTTCTCAG |
|  | Reverse | CCATGTTGGCACTGTTGTTG |
| CD24 | Forward | GGTTGTCTCCTGCGACTTCA |
|  | Reverse | TGGTCCAGGGTTTCTTACTCC |
| LAG3 | Forward | GCGGGGACTTCTCGCTATG |
|  | Reverse | GGCTCTGAGAGATCCTGGGG |
| CTLA4 | Forward | CATGATGGGGAATGAGTTGACC |
|  | Reverse | TCAGTCCTTGGATAGTGAGGTTC |
| BTLA | Forward | CATCTTAGCAGGAGATCCCTTTG |
|  | Reverse | GACCCATTGTCATTAGGAAGCA |
| PDCD1 | Forward | CCAGGATGGTTCTTAGACTCCC |
|  | Reverse | TTTAGCACGAAGCTCTCCGAT |
| HAVCR2 | Forward | TTGGACATCCAGATACTGGCT |
|  | Reverse | CACTGTCTGCTAGAGTCACATTC |

GAPDH, Glyceraldehyde-3-Phosphate Dehydrogenase; CD24, Cluster of Differentiation 24; LAG-3, Lymphocyte-activation gene 3; CTLA-4, cytotoxic T lymphocyte-associated antigen-4; BTLA, B and T lymphocyte attenuator; PDCD1, programmed cell death protein-1;HAVCR2, hepatitis a virus cell receptor 2.

Supplementary Table 3. Correlation of CD24 mRNA expression and clinical prognosis in liver cancer with different clinicopathology factors by Kaplan-Meier plotter.

| Clinicopathological characteristics | Overall survival (n=364) | | | Progression-free survival (n=370) | | |
| --- | --- | --- | --- | --- | --- | --- |
|  | N | Hazard ratio | P-value | N | Hazard ratio | P-value |
| SEX |  |  |  |  |  |  |
| Female | 121 | 2.52 (1.38-4.59) | 0.0018 | 121 | 2.74 (1.61-4.67) | 0.0001 |
| Male | 250 | 1.74 (1-3.02) | 0.0046 | 250 | 1.49 (1.01-2.21) | 0.0436 |
| Alcohol consumption |  |  |  |  |  |  |
| Yes | 117 | 1.57 (0.8-3.09) | 0.1867 | 117 | 2.3 (1.32-4.01) | 0.0025 |
| None | 205 | 1.99 (1.23-3.21) | 0.0043 | 205 | 1.91 (1.26-2.88) | 0.0018 |
| Hepatitis virus |  |  |  |  |  |  |
| Yes | 153 | 0.74 (0.38-1.42 | 0.3608 | 153 | 1.78 (1.1-2.88) | 0.0176 |
| None | 169 | 2.09 (1.27-3.44) | 0.0031 | 169 | 2.29 (1.44-3.65) | 0.0003 |
| Stage |  |  |  |  |  |  |
| 1 | 171 | 1.57 (0.81-3.02) | 0.1753 | 171 | 1.76 (1.05-2.97) | 0.0307 |
| 2 | 86 | 0.41 (0.18-0.96) | 0.0338 | 86 | 1.49 (0.76-2.9) | 0.2393 |
| 3 | 85 | 2.38 (1.21-4.7) | 0.0099 | 85 | 1.61 (0.88-2.95) | 0.1194 |
| 4 | 5 | - |  | 5 | - |  |
| Grade |  |  |  |  |  |  |
| 1 | 55 | 2.26 (0.81-6.26) | 0.1088 | 55 | 2.41 (1-5.77) | 0.0426 |
| 2 | 177 | 2.29 (1.32-3.95) | 0.0023 | 177 | 2.1 (1.34-3.32) | 0.001 |
| 3 | 122 | 0.59 (0.32-1.07) | 0.0805 | 122 | 1.45 (0.88-2.39) | 0.1379 |
| 4 | 12 | - |  | 12 | - |  |
| AJCC-T |  |  |  |  |  |  |
| 1 | 181 | 1.6 (0.85-3.01) | 0.1401 | 181 | 1.94 (1.16-3.23) | 0.0097 |
| 2 | 94 | 0.54 (0.26-1.15) | 0.1058 | 94 | 1.54 (0.82-2.9) | 0.1766 |
| 3 | 80 | 2.54 (1.31-4.92) | 0.0044 | 80 | 1.66 (0.88-3.12) | 0.1159 |
| 4 | 13 | - |  | 13 | - | - |
| Vascular invasion |  |  |  |  |  |  |
| micro | 93 | 3.02 (1.04-8.81) | 0.0332 | 93 | 1.81 (1.02-3.23) | 0.0401 |
| macro | 16 | - |  | - |  | - |
| none | 205 | 1.83 (1.05-3.18) | 0.0294 | 205 | 1.71 (1.08-2.72) | 0.0217 |

Supplementary Table 4. Correlation analysis between CD24 and gene markers of immune cells in TIMER.

| Description | Gene markers | LIHC | | | |
| --- | --- | --- | --- | --- | --- |
|  |  | None | | Purity | |
|  |  | Cor | P | Cor | P |
| B cell | CD19 | 0.1870954432444 | *** | 0.1737179520622 | ** |
|  | CD79A | 0.2091488853231 | *** | 0.1885861572335 | *** |
|  | CD22 | 0.3436062165926 | *** | 0.3221205130596 | *** |
|  | CD40 | 0.0100120553559 | 0.8475 | 0.0005602532168 | 0.9917 |
| T cell (general) | CD3D | 0.2725473930587 | *** | 0.2580559561984 | *** |
|  | CD3E | 0.2188044752865 | *** | 0.2100209557783 | *** |
|  | CD2 | 0.2291311918823 | *** | 0.2183788361552 | *** |
| CD8 + T cell | CD8A | 0.1589156759577 | ** | 0.1428620380794 | ** |
|  | CD8B | 0.1143529503552 | * | 0.0890294766680 | 0.0988 |
| Monocyte | CD86 | 0.3026115237923 | *** | 0.3060117638007 | *** |
|  | CSF1R | 0.2100263901846 | *** | 0.2057137177505 | *** |
| TAM | CCL2 | 0.1780839268971 | *** | 0.1453966741056 | ** |
|  | CD68 | 0.2525575919368 | *** |  |  |
|  | IL10 | 0.1989533701843 | *** | 0.1845466654810 | *** |
| M1 macrophage | IRF5 | 0.2755512608511 | *** | 0.2708930026807 | *** |
|  | PTGS2 | 0.3128585330370 | *** | 0.3134044802368 | *** |
|  | NOS2 | 0.0415637695923 | 0.4247 | 0.0396418460131 | 0.4630 |
| M2 macrophage | CD163 | 0.0492784469128 | 0.3439 | 0.0296445508388 | 0.5831 |
|  | VSIG4 | 0.1449732925692 | ** | 0.1362604712320 | * |
|  | MS4A4A | 0.1111083172556 | * | 0.0987737458866 | 0.0669 |
| Neutrophils | CEACAM8 | 0.1091410347138 | * | 0.1108022350535 | * |
|  | ITGAM | 0.2994703163297 | *** | 0.2929179394849 | *** |
|  | CCR7 | 0.1474116320914 | ** | 0.1223521820419 | * |
| Nature killer cell | KIR2DL1 | -0.0498764698758 | 0.3380 | -0.0610142523539 | 0.2584 |
|  | KIR2DL3 | 0.0253976498354 | 0.6258 | 0.0161359046969 | 0.7652 |
|  | KIR2DL4 | 0.0176807330479 | 0.7343 | -0.0010859412992 | 0.9840 |
|  | KIR3DL1 | -0.0572813944123 | 0.2711 | -0.0664331516335 | 0.2184 |
|  | KIR3DL2 | 0.0398175650882 | 0.4444 | 0.0295876715685 | 0.5839 |
|  | KIR3DL3 | -0.0489945831559 | 0.3467 | -0.0424850455769 | 0.4315 |
|  | KIR2DS4 | -0.0338654414039 | 0.5155 | -0.0333483381550 | 0.5370 |
| Dendritic cell | HLA-DPB1 | 0.2066071810442 | *** | 0.1910904007256 | *** |
|  | HLA-DQB1 | 0.1402427990986 | ** | 0.1104613958565 | * |
|  | HLA-DRA | 0.1782063604340 | *** | 0.1636352719582 | ** |
|  | HLA-DPA1 | 0.1954149697911 | *** | 0.1907036027764 | *** |
|  | CD1C | 0.2325935588867 | *** | 0.2045533814950 | *** |
|  | NRP1 | 0.2516838723777 | *** | 0.2368183793405 | *** |
|  | ITGAX | 0.3472452454193 | *** | 0.3492495043335 | *** |

**p <* 0.05, ***p* < 0.01, ****p* < 0.001, *****p* < 0.0001.

**Supplemental Figures**

Supplementary figure 1


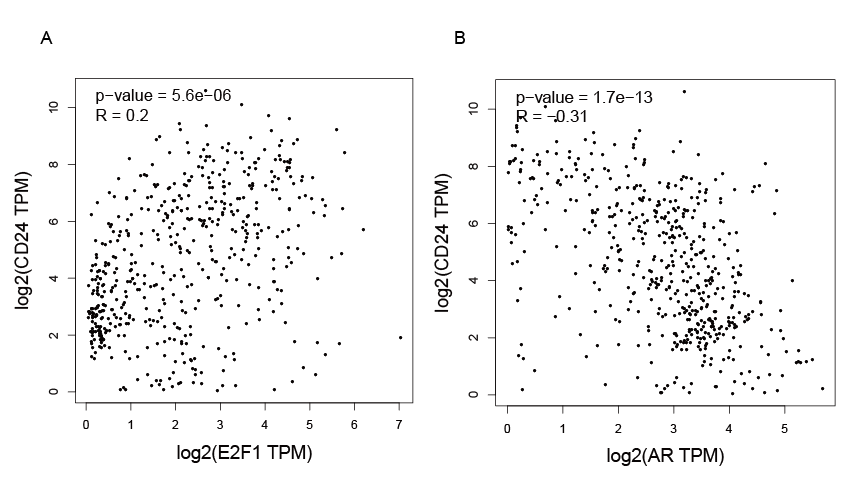


Fig.S1 Correlation analysis of AR and E2F1 with CD24. A-B Correlation analysis of AR and E2F1 with CD24 by GEPIA database.

Supplementary figure 2


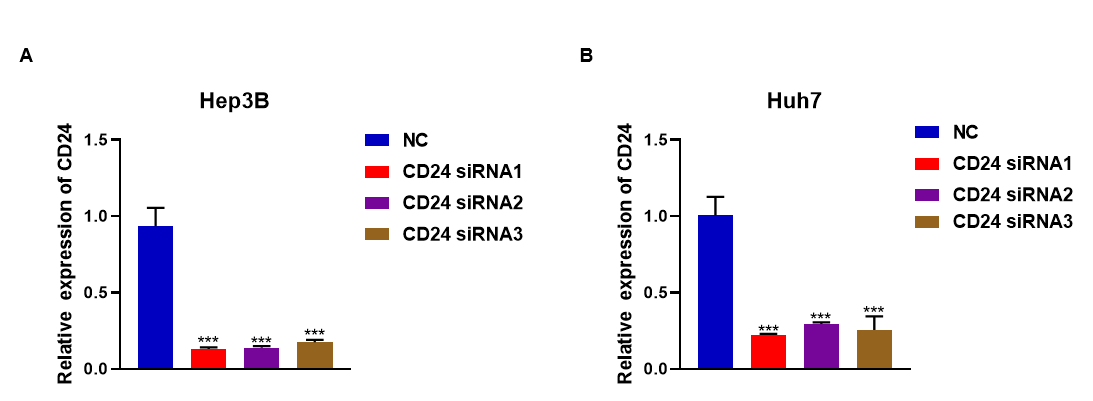


Fig.S2 The transfection efficiency of CD24 siRNAs in Hep3B and Huh7 cells. A-B. qRT-PCR analysis of CD24 levels in Hep3B and Huh7 cells after transfection with CD24 siRNA. ^***^*p <* 0.001.
